# Synanthropic and Wild Animals as Sentinels of Zoonotic Agents: A Study of *Leptospira* Genotypes Circulating in Northeastern Italy

**DOI:** 10.3390/ijerph20053783

**Published:** 2023-02-21

**Authors:** Elisa Mazzotta, Laura Bellinati, Cristina Bertasio, Maria Beatrice Boniotti, Laura Lucchese, Letizia Ceglie, Federico Martignago, Stefania Leopardi, Alda Natale

**Affiliations:** 1 Istituto Zooprofilattico Sperimentale delle Venezie, 35020 Legnaro, Italy; 2 National Reference Centre for Animal Leptospirosis, Istituto Zooprofilattico Sperimentale della Lombardia ed Emilia Romagna “Bruno Ubertini”, 25121 Brescia, Italy

**Keywords:** *Leptospira*, synanthropic animals, environmental reservoirs, multi-locus sequence typing, real-time PCR

## Abstract

Leptospirosis is an infectious disease widely reported in veterinary practice and a worldwide zoonosis. In Northeastern Italy, different serogroups and genotypes of *Leptospira* have been described in ill dogs, the most commonly detected being Icterohaemorragiae (ICT) ST 17, Australis (AUS) ST 24 and ST 198, Pomona (POM) ST 117 and ST 289, and Sejroe (SEJ) ST 155. However, there is little information available on the environmental exposure to *Leptospira* of wild and synanthropic animals. The aim of this study was to identify the circulating genotypes in potential reservoirs to fill this gap of knowledge. Between 2015 and 2022, 681 animal carcasses collected by the Public Veterinary Service were analyzed for *Leptospira* with a real-time PCR-based screening test, while positive samples were genotyped by multi-locus sequence typing analysis. To carry out our study, we tested 330 hedgehogs, 105 red foxes, 108 Norway rats, 79 mice, 22 coypus, 10 bank voles, 13 grey wolves, 5 common shrews and 9 greater mouse-eared bats. Five sequence types (STs) common in dogs were also found in wild animals: ST 24, ST 198, ST 17 and ST 155 in hedgehogs, ST 17 and ST 24 in foxes, ST 17 in rats, ST 17 and ST 155 in mice, and ST 117 in a wolf. In addition, to the best of the authors’ knowledge, this is the first Italian report of SEJ ST 197 in a bank vole. Furthermore, this study described a previous survey conducted in 2009 on coypus (30 animals from the province of Trento and 41 from the province of Padua), referring to a serological positivity (*L*. Bratislava) without any molecular detection of *Leptospira*. This study on *Leptospira* in synanthropic and wild animals highlighted the importance of increasing our epidemiological knowledge of leptospirosis and its zoonotic risks.

## 1. Introduction

Leptospirosis is a major zoonotic disease worldwide, with a significant impact on both human and animal health [1,2]. The disease is caused by pathogenic spirochete bacteria of the genus *Leptospira*, which consists of 22 known species (pathogenic, intermediate, and saprophytic), including more than 300 serovars [3]. Twelve novel species of *Leptospira* have recently been isolated from tropical soils, suggesting a highly unexplored biodiversity in the genus [4].

Pathogenic *Leptospira* spp. colonize the proximal renal tubules of reservoir hosts that remain asymptomatic but can shed leptospires via urine and infect the external environment where, in favourable conditions, they can survive for long periods, causing contamination of surface water, soil, and muddy areas. Human infection can occur through direct contact with infected animals or, more frequently, through contacts with the contaminated environment. Human cases of leptospirosis have traditionally been associated with outdoor occupational activities, particularly in those cases when contacts with backwaters and domestic or synanthropic animals (e.g., dogs, rats, cattle, pigs) [5,6] occurred; however, sometimes the role of domestic animals, such as dogs, as direct vehicles of *Leptospira* infection may have been overstated [7]. In developed countries, leptospirosis has recently become a disease more related to recreational activities with exposure to fresh water (canoeing, swimming, and canyoning) [8,9]. Furthermore, leptospirosis infection in humans appears to be associated with natural disasters, particularly when floods occur [10,11].

Among wild and synanthropic species, rats and small rodents are considered the most common carriers and reservoirs of the infection [12]. Evidence of *Leptospira* infection has been reported in European wild carnivore species such as red fox (*Vulpes vulpes*), pine marten (*Martes martes*), stone marten (*Martes foina*), badger (*Meles meles*), lynx (*Lynx lynx*), brown bear (*Ursus arctos*), and grey wolf (*Canis lupus*) [13,14,15]. Moreover, there is growing evidence that bats (order Chiroptera) are infected by very different leptospires, especially in tropical regions with a high abundance of bat species [16], whereas in Europe, information is scarce.

*Leptospira* susceptibility and infection have also been considered in a European invasive rodent species, such as the coypu [17,18] (*Myocastor coypus* Molina, 1782), a medium-sized rodent typical of aquatic environments, originating from South America and imported in Europe at the beginning of the twentieth century for commercial breeding purposes. Negligence in the early containment of the animals resulted in repeated releases of this exotic species into the environment, which led to the naturalization of the coypu in Italy and in many other European countries, where this species has created extensive damage to ecosystems. Moreover, the coypu has been recognized as a possible source of transmission of numerous bacterial and viral diseases, although its specific role as a reservoir for *Leptospira* is still under investigation [19,20].

Considering the ecological role of synanthropic animals in the ecosystem, their habit, nature and range, they may be crucial for the environmental circulation of *Leptospira* spp.: recent studies in fact report that *Leptospira* spp. positivity in wildlife could be different according to the season [21], geographic area, and yearly rainfall [22,23].

The aim of this study was to report previous results of *Leptospira* seroprevalence in a largely diffuse rodent, such as the coypu, and to explore the potential epidemiological role of synanthropic and wild small mammals, such as hedgehogs (*Erinaceus europaeus* Linnaeus, 1758), red foxes (*Vulpes vulpes* Linnaeus, 1758), Norway rats (*Rattus rattus, Fischer*, 1803), house mice (*Mus musculus* Linnaeus, 1758), common shrew (*Sorex araneus* Linnaeus, 1758), bank voles (*Myodes glareolus* Schreber, 1780), and the greater mouse-eared bat (*Myotis myotis*). This survey attempted to better understand the role of these species as sentinels or reservoirs of pathogenic leptospires, focusing on the species having a major epidemiological role in the environmental maintenance of leptospirosis and its transmission to humans.

## 2. Materials and Methods

### 2.1. Sampling

#### 2.1.1. Myocastor Coypus Eradication Action Policy: Sampling Period 2009

Over a 12-month period in 2009, 30 coypus from the province of Trento and 41 animals from the province of Padua were enrolled in the present study. The animals were captured and subsequently suppressed as part of the culling campaign conducted by the Trentino Hunters’ Association and the Padua Provincial Police Force. All samples were collected from rodents legally killed for population control; therefore, this study did not involve the deliberate additional killing of animals. All procedures for population control were in compliance with the ethical standards of relevant national and European regulations on the care and use of animals. The captured individuals were immediately euthanized. Subsequently, the rodents were necropsied for collection of a cardiac clot, urine sample and kidney tissue. Tissues and urine samples were immediately stored at −20 °C until molecular analysis.

The cardiac clots were submitted to a microagglutination test (MAT), according to the World Organization for Animal Health (WOAH) method (Chap 3.1.12) [3]. The antigen panel included 8 serogroups and 9 serovars and was distributed by the Italian Reference Centre for Animal Leptospirosis, Istituto Zooprofilattico Sperimentale della Lombardia ed Emilia Romagna (IZSLER) for the routine diagnostic MAT. Serum samples obtained by the cardiac clot, were pre-tested at the final dilution of 1:100. Serum with 50% agglutination was further analyzed to determine an endpoint using dilutions of serum beginning at 1:100 through to 1:6400. Serum samples with the widely accepted minimum significant titre of 1:100 (reciprocal of the final dilution of serum with 50% agglutination) were assessed as positive.

*Leptospira* isolation was attempted from urine and kidney samples, as previously described [24]. Kidney tissue and urine samples were also screened for the presence of *Leptospira* spp. through an end-point PCR assay targeting a 423 bp fragment within the region of the lipL32 gene, conserved among pathogenic *Leptospira* serovars [25].

#### 2.1.2. Synanthropic and Wild Animals Public Veterinary Survey: Sampling from 2015 to 2022

Passive surveillance of synanthropic and wild animals was carried out on animal carcasses collected by the Public Veterinary Service during the routine surveillance program, therefore no deliberate killing of animals was contemplated for this sampling. Between 2015 and April 2022, 681 synanthropic and wild animal carcasses were submitted for analyses (i.e., 9 greater mouse-eared bats, 10 bank vole, 105 red foxes, 5 common shrew, 330 hedgehogs, 79 house mice, 108 Norway rats, 22 coypus, 13 grey wolves). Kidney tissue or urine were collected and further investigated for the presence of pathogenic *Leptospira*. Epidemiological data (location, time of sampling) were recorded for each sample; however, due to the study design, no additional information was available to be used for a complete spatial and modelling analysis.

### 2.2. Molecular Analysis

Kidney tissue samples were homogenized at a 1:10 dilution in 600 µL of sterile PBS, with TissueLyser II (QIAGEN, Hilden, Germany). Two mL of urine samples were centrifuged at 12,000 g for 20 min at 4 °C and the pellet was re-suspended in 0.2 mL of sterile PBS, in order to possibly concentrate the leptospires and increase the yield of DNA. Given the poor cellular matrices, 20 µg of a poly-A carrier (Sigma-Aldrich, St. Louis, MO, USA) was added to each urine sample to increase the recovery efficiency of nucleic acids.

DNA isolation from 100 µL of tissue homogenate or urine pellet, was performed after a pre-lysis treatment with 2.5 μL of lysozyme (10 mg/mL in 10 mM Tris-HCl, pH 8.0) for 15 min at +37 °C. The DNA extraction was performed on the KingFisher™ Flex Purification System (Life Technologies, Carlsbad, CA, USA) platform using the ID Gene^®^ Mag Universal Extraction Kit (IDvet, Grabels, France), in accordance with the manufacturer’s instructions. Each DNA extraction session included a negative process control (water).

The presence of pathogenic species of *Leptospira* was searched by means of a screening real time PCR (qPCR) targeting a 87 bp fragment that corresponded to a portion of the gene encoding the 16S rDNA [26]. The qPCR was performed in a 25 µL final volume, containing 3 µL of extracted DNA, 12.5 µL of 2× Path-ID™ qPCR Master Mix (Thermo Fisher Scientific, Waltham, MA, USA), 300 nM of each primer, and 100 nM of a 5′ 6-carboxyfluorescein (FAM)–3′-tetramethylrhodamine (TAMRA) probe. The amplification assay included a negative control (water), a negative bacterial genomic control (DNA of *Leptospira* biflexa serovar Patoc), and a positive control (DNA of *L. interrogans* serovar Icterohaemorrhagiae). The assay was performed under the following thermal conditions: a holding step at 95 °C for 10 min and 40 cycles at 95 °C for 15 s and 60 °C for 60 s. Samples with cycle threshold (Ct) < 38 were considered positive. Samples having Ct values within the 38–40 range were considered doubtful, whereas samples having no FAM fluorescence signal or with Ct ≥ 40 were considered negative.

### 2.3. Genotyping

DNA of positive samples were referred to the IZSLER National Reference Centre for Leptospirosis for the genomic characterization with Multi-locus Sequence Typing (MLST) technique. 

The genotyping was attempted in all qPCR positive samples by using the 7-loci scheme proposed by Boonsilp in 2013 [27], which is based on the housekeeping genes *glmU, pntA, sucA, tpiA, pfkB, mreA* and *caiB*, as previously described [24]. The sequence analysis and the identification of the sequence types were performed using Bionumerics software ver. 7.6 (Applied Math, Biomerieux, Sint-Martens-Latem, Belgium) through the query of the Bacterial Isolate Genome Sequence database (BIGSdb) available on the *Leptospira* multilocus sequence typing (MLST) website (https://pubmlst.org/leptospira/) sited at the University of Oxford [28]. Comparisons between the STs found and those present in BIGSdb associated to completely typed isolates were used to deduce the species, the serotype, and (sometimes) the serovar of the *Leptospira* being tested.

### 2.4. Data Anaylisis

#### Statistical Analysis

To identify the factors that may be significantly correlated with *Leptospira* spp. positivity, Bayesian generalized linear mixed models (GLMM) were used. These models were adapted by considering the following fixed effects: the origin of the samples (province), the species and the season of collection. To assess any correlation between *Leptospira* positivity in synanthropic and wild animals and seasonality, each sample was placed in a seasonal time slot, as follows: in spring, if sampled from March to May; in summer, if collected from June to August; in autumn, if collected from September to November; and in winter, if collected from December to February. Analyses were performed using the R software package “blme″ (R Development Core Team 2013) with binomial error (logit-link function) using the year of collection as a random effect [29].

Data about the geographic origin of the samples (province) were depicted in a map which displayed the distribution of positive samples and genotypes, in order to make the spatial distribution easy to understand. The map was created using the open-source software QGIS 3.16.

## 3. Results

### 3.1. Sampling and Molecular Analysis

#### 3.1.1. Myocastor Coypus Eradication Action Policy: Sampling Period 2009

In 2009, a total of 30 coypus were collected in the province of Trento and 41 animals in the province of Padua. The serological examination among the coypus enrolled from the Trento province reported 2/30 animals (6.7%, 95%CI 0.8–22.1) as seropositive, both with antibodies for *L*. Australis serovar Bratislava (titres of 1:200 and 1:1600). From the province of Padua, 24/41 animals (58.5%, 95%CI 42.1–73.7) resulted seropositive to different serovars: 21/41 (51.2%, 95%CI 35.1–67.1) reported serological positivity for *L*. Australis serovar Bratislava (antibodies titres ranging from 1:100 a 1:3200), 2/41 coypus (4.9%, 95%CI 0.006–0.165) had low antibodies titres against *L*. Icterohaemorragiae serovar Icterohaemorragiae (titres 1:100 and 1:200), 1/41 subject (2.4%, 95%CI 0.1–12.9) reported a low antibody positivity against *L*. Icterohaemorragiae serovar Copenhageni (1:100) (Table 1) The molecular analysis performed both on the kidney tissue and urine samples were negative for all samples and no *Leptospira* spp. was isolated, therefore genotyping was not possible.

#### 3.1.2. Synanthropic and Wild Animals: Sampling from 2015 to 2022

Between January 2015 and April 2022, the Public Veterinary services of Northeastern Italy collected 681 animal carcasses, belonging to different species of synanthropic and wild animals: 9 greater mouse-eared bats, 10 bank vole, 105 red foxes, 5 common shrew, 330 hedgehogs, 79 house mice, 108 Norway rats, 22 coypus, 13 grey wolves. For each animal, a molecular investigation through qPCR was performed on the kidney tissue or urine samples.

The overall prevalence of pathogenic *Leptospira* spp. in each enrolled animal is reported in Table 2. A high prevalence was recorded in coypus (40.9%, *n* = 9/22), bank voles (30%, *n* = 3/10), Norway rats (23%, *n* = 25/108), red foxes (21%, *n* = 22/105), hedgehogs (17.6%, *n* = 58/330). Samples from house mice (11.4%, *n* = 9/79), grey wolves (7.7%, *n* = 1/13), greater-mouse eared bats (11%, *n* = 1/9) and shrews (20%, *n* = 1/5) show a lower prevalence.

**Figure 1:**
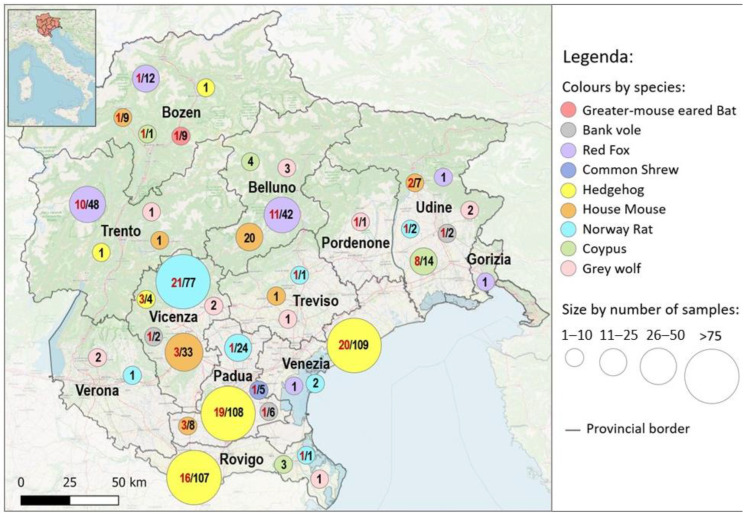
Synanthropic and wild animals analyzed for *Leptospira* spp. geographically mapped. The size of each dot changes according to the total number of samples examined, while the colors represent the different animal species, as indicated above. Black numbers indicate the total samples of each species analysed per province, while red numbers highlight the positive samples, when present.

Figure 1 reports the geographical localization of the collected samples and the qPCR results from each site.

Samples of grey wolves, bank voles, and common shrews were consistently distributed between the different collection sites, while red foxes were mainly concentrated in the provinces of Belluno, Trento and Bolzano, in the alpine and pre-alpine region. In contrast, hedgehogs were mostly obtained from the provinces of Venice, Padua and Rovigo, and the Norway rat from the provinces of Padua and Vicenza. These provinces are located in the geographical areas of the Po Valley. Most of the positive coypus samples were collected from a single geographical site and within a short period of time (7–21 days). The nine mouse-eared bats were collected from a single colony and only one tested weakly positive.

### 3.2. Genotyping

DNA of positive samples was submitted to the genomic characterization with MLST technique to deduce the species, the serotype and/or the serovar of the *Leptospira* tested. Both the complete and the partial MLST profiles were reported and placed in a corresponding ST. For allelic number and ST identification, assembled and trimmed sequences were queried against the Bacterial Isolate Genome Sequence Database (BIGSdb) available on the *Leptospira* MLST website (https://pubmlst.org/leptospira/, accessed on 1 December 2022) sited at the University of Oxford [28]. Comparisons between the STs found and those present in BIGSdb as reference isolates were used to deduce the species of the *Leptospira* being tested. To perform comparisons among historical serological studies (where serovars and serogroups were defined) and genotyping data (where species and genomic profiles were defined), we chose to assign to each identified ST a classification at the serogroup and serovar levels obtained from BIGSdb, knowing that this information was deduced and did not result from active serological typing.

*Leptospira* serogroups such as Australis and Icterohaemorrhagiae have been reported, as well as fewer other different serogroups (Sejroe, Javanica and Ballum).

As described in Table 3, an identifiable MLST profile was obtained only for 83 positive samples from the 129 animals found positive for pathogenic *Leptospira* spp. with qPCR, 

The overall prevalence in the sampled animals was 18.9% (CI95% 16.1–22.1), no statistically significant difference in *Leptospira* positivity was found between the different sampled species. The most represented serogroup among the positive samples was Australis (53%; *n* = 44/83, 95%CI 42.2–63.7), reporting positivity both for ST 24 (34.9%; *n* = 29/83, 95%CI 24.6–45.2) and ST 198 (18.1%, *n* = 15/83, 95%CI 9.7–26.4). Among the species, ST 24 was reported in hedgehogs (28.9%; *n* = 24/83), house mouse (1.2%; *n* = 1/83) and red foxes (4.8%; *n* = 4/83). Furthermore, ST 198 was detected exclusively in hedgehogs (18.1%; *n* = 15/83).

*L. interrogans* Icterohaemorragiae ST 17 demonstrated an overall prevalence of 37.5% (*n* = 31/83, 95%CI 26.7–47.8) among the positive samples: ST 17 was reported in Norway rats (26.5%; *n* = 22/83), in hedgehogs (3.6%; *n* = 3/83), house mice (3.6%; *n* = 3/83) and red foxes (1.2%; *n* = 1/83). *L. borgpetersenii* Javanica ST 146 was reported in 4.8% of positive animals (*n* = 4/83, 95%CI 0.21–9.43), specifically in one hedgehog (1.2%; *n* = 1/83), one house mouse (1.2%; *n* = 1/83) and two red foxes (2.4%; *n* = 2/83). *L. borgpetersenii* Sejroe reported positivity for ST 155 (2.4%; *n* = 2/83, 95%CI 0.0–5.7) in one hedgehog (1.2%; *n* = 1/83) and in one house mouse (1.2%; *n* = 1/83), respectively. Moreover, a positivity for *L. borgpetersenii* Sejroe ST 197 was reported in a bank vole (1.2%; *n* = 1/83, CI95 0.0–3.5). *L. kirschneri* Pomona ST 117 was reported in one grey wolf (1.2%; *n* = 1/83, CI95 0.0–3.5).

**Figure 2:**
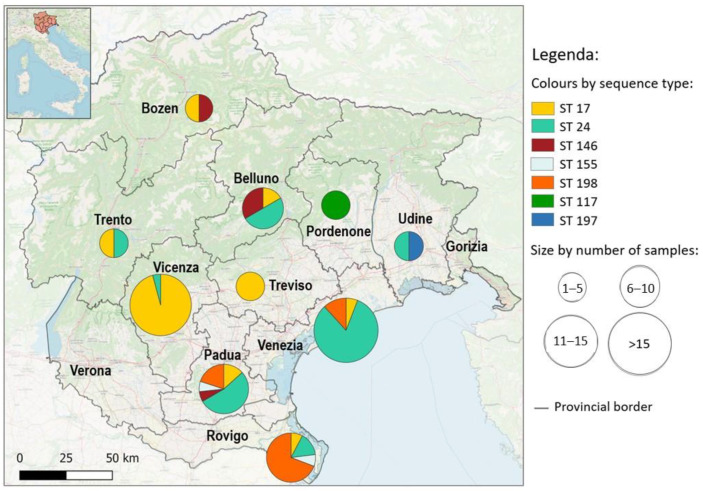
Sequence-Type (ST) geographical distribution. The size of each pie chart represents the total number of samples, the colors represent the different Sequence Type found for each Italian province analyzed.

Figure 2 shows the distribution of the identified STs in the provinces included in the study. The small sample size could be a limiting factor, reason being that genotypes are not homogeneously represented in all provinces. It is interesting to note that in the provinces of Venice, Padua and Rovigo, territories that gave a conspicuous number of hedgehogs that tested positive for *Leptospira* at molecular analysis, variability in STs is evident. ST 24 is more represented in hedgehogs from Padua and Venice, whereas ST 198 is more frequent in hedgehogs from Rovigo.

The province of Udine was found to have a fourfold higher prevalence of positivity to *Leptospira* spp. than the other provinces (odds ratio: 4.01, CI95% 1.1–14.2, *p* < 0.05), but this figure is difficult to relate in a standard model, as the sampling was carried out following the reporting of an outbreak of Leptospirosis (geographically close sites and short period of time).

### 3.3. Evaluation of Seasonality as an Effect on Leptospira Positivity

The differences in prevalence of the number of animals positive to *Leptospira* spp. in the four seasons were calculated for the overall population of the study, and individually for the species with the highest number of collected samples (hedgehog, house mouse, Norway rat and red fox). A GLMM was used to determine the seasonal differences in *Leptospira* positivity with the year and province of collection as random effects, the p values of the Wald chi-square tests for the GLMMs for each species is shown in Table 4.

The analysis of *Leptospira* spp. prevalence among the wild and synanthropic animal population revealed no significant correlation with the season, except in the model represented a statistically significant fluctuation in the seasonal prevalence of infection with pathogenic *Leptospira* (*p* < 0.05), with a higher prevalence in summer.

## 4. Discussion

According to previous studies in the literature, serological studies of *Leptospira* in wild rodents in Europe report a high seroprevalence (75–86%), and the most represented serogroups are Australis, Autumnalis, Icterohaemorragiae, Grippotyphosa, Panama, and Sejroe. [14]. Conversely, molecular analyses of pathogenic strains of *Leptospira* in European small mammals reported a different prevalence of *Leptospira* DNA detection [30,31]. It is not clear how long small mammals (i.e., mustelids, small rodent, wild carnivores) can shed *Leptospira* after infection, hence their epidemiological role needs to be evaluated in comparison with multiple factors (i.e., ecology, population density, environment, urbanization) [30,32]. In addition, the biological balance between pathogenic leptospires and reservoir hosts of infection is not well defined: particularly, the host’s factors (species, immune response, resistance factors) that facilitate persistent renal colonization in synanthropic and wild animals, are still unclear [33].

### 4.1. Myocastor Coypus

Many surveys worldwide [13,34,35] report that all rodents, but mostly rats and mice, show serologic positivity for *Leptospira*, with a prevalence ranging from 50 to 90%, whereas coypus did not show a high prevalence of *Leptospira* in Europe [30,36]. This study reported a significant seroprevalence in an active surveillance survey in coypus in 2009, with serogroup *Australis* as the most represented (*n* = 26/71, 36.6%), but none of the animals tested positive after molecular screening. Similar results were reported in a study conducted in Northwestern Italy, in which *L.* Australis/Bratislava was the most represented serogroup among coypus [19], and in France [37]. Different assumptions have been made to explain these findings, including the ability of leptospires infecting coypus to adapt to their host, the occurrence of several consecutive infections or the exposure to the pathogenic *Leptospira* with an increased antibodies reaction by cross- reaction [36].

We performed molecular analyses on the same species sampled from the coypu population between 2015 and 2022 during a passive surveillance program, reporting high prevalence of *Leptospira* (40.9%), although the value obtained in the qPCR analysis indicates a low bacterial load. According with studies in the previous literature [37], this high molecular prevalence may be due to the exposure to a contaminated habitat; indeed, most of the positive animals came from the same geographical site and were collected within the same period.

Previous studies reported that coypus seem to be a less efficient maintenance hosts rather than other species (i.e., rats, muskrats) [36], although precise data on the impact of coypu on environmental contamination and the size of the coypu population in northeastern Italy are not available. It is commonly suspected that the uncontrolled reproduction of this species may increase the wild reservoirs of *Leptospira*. The present study highlights the need to further investigate the epidemiologic role of coypus as a non-autochthonous rodent in a *Leptospira*-contaminating environment (rate and duration of leptospires excretion) and transmission to other susceptible hosts.

### 4.2. Synanthropic and Wild Animals

The most shared and accepted definition of reservoir is “a population which is chronically infested with the causative agent of a disease and can infect other populations” [38]. The condition of the reservoir and/or carrier for pathogenic *Leptospira*, as well as some other infections given by multi-host pathogens, is not clearly defined, mainly due to the scarce epidemiological and ecological information. In this study, we reported a broad exposure and geographical distribution of *Leptospira* spp. in synanthropic species and wildlife in northeastern Italy. In agreement with studies in the previous literature [30,31], this study reported the presence of pathogenic *Leptospira* spp. in Norway rats (23%), bank voles (30%), house mice (11.4%), a common shrew and in hedgehogs (17.6%). Among the carnivourous, one grey wolf reported positively for *Leptospira* and red foxes were also found to be positive with a prevalence of 21%. In addition, we detected a pathogenic *Leptospira* spp. positivity in a greater-mouse eared bat, but the low bacterial load did not allow the identification of the strain.

MLST molecular techniques used in this study provided a specific and unique identification of the strains of *Leptospira* infecting the animal host, providing a helpful and powerful tool in investigating the epidemiology of leptospirosis. In this study, the association of *Leptospira* ST with small mammals showed different patterns: serogroups Icterohaemorragiae, Sejroe, Javanica and Australis were detected in multiple host species (Table 2). The overrepresentation of *L*. Icterohaemorragiae ST 17 in Norway rats has led to the identification of this species as the main environmental reservoir and carrier, also according to studies in the previous literature [34]. European hedgehogs were more frequently infected by *L.* Australis than any other small mammal species, showing positivity to both ST 24 and ST 198: similar results were previously reported [39], suggesting that this species may represent the reservoir and carrier of this ST in Europe.

Furthermore, we reported *L*. Australis ST 24, and Icterohaemorragiae ST 17 in red foxes, in agreement with the serovars observed in a recent study on the *Leptospira* seroprevalence of red foxes conducted in other European countries: central and eastern Poland (26.3%) [40], Croatia (31.3%) [41], Spain (47.1%) [42] and Norway (9.9%) [43]. Significantly, the identification of *L.* Javanica ST 146 in this species suggests the broadening of the host spectrum of this serogroup among wild carnivores. 

To the best of the authors’ knowledge, this is the first Italian report of *L. borgpetersenii* Sejroe ST 197 in a bank vole. According to our results, ST 197 had already been reported in bank voles in Europe [23]. The identification of this serogroup, which is pathogenic for multiple species, underlies the significance of this species as reservoirs for *Leptospira* spp. and sources of infection for humans and livestock. As supposed for other *Leptospira* species, the detection of ST 197 in different geographical regions may suggest the development of new patterns of diffusion or transmission, questioning the ‘‘one pathogen-one carrier’’ hypothesis [30].

As previously reported in greater detail [15], in this study we identified a positivity to *L*. kirschneri, serogroup Pomona, serovar Mozdok (ST 117) in a grey wolf. This serogroup has also been reported among wild boars (*Sus scrofa*) with a seroprevalence of 4.18% in central Italy [44], 45% in Slovenia [45] and 7% in Spain [46]. In eastern Croatia *L*. *kirschneri*, serogroup Pomona, serovar Mozdok was the most frequently isolated *Leptospira* in small rodents [47], and sequence type ST 117 was also detected in small mammals in Germany [31]. In addition, serogroup Pomona has been reported as one of the most-represented among stray dogs (40.8%) [48]. Therefore, the positivity in large carnivores could be connected to the prey–predator epidemiology [15] or to a domestic–wild ecological pattern. Furthermore, specific molecular techniques, such as multi-locus sequence typing (MLST) [27] and multiple loci variable-number tandem repeat analysis (MLVA) [49], can provide a specific and unique codification for the identified *Leptospira* through the analysis of specific fragments of bacterial loci. Through the assignment of sequence types (STs), MLST allows for objective comparisons to be made among *Leptospira* strains infecting the same host in different geographic regions or different host species within the same geographic area, thus providing a helpful and powerful tool to investigate the epidemiology of leptospirosis. As previously reported, these research teams described the circulation of *Leptospira* among domestic dogs: the most reported STs belong to *L. interrogans* Icterohaemorragiae (serovar Icterohaemorrhagiae or Copenhageni, ST 17), *L. interrogans* Australis (serovar Australis, ST 198 and serovar Bratislava or Jalna, ST 24). *L. kirschneri* serogroup Pomona serovar Mozdok (ST 117 and ST 289) and *L*. *borgpetersenii* serogroup Sejroe (ST 155) have also been identified in dogs with clinical leptospirosis [50]. To date, there is no evidence that wolf populations may represent a reservoir of infection; nevertheless, further surveillance and epidemiological evaluations will be crucial to better understand both *Leptospira* strains distribution and the relationship at the wildlife/domestic animals/human interface.

### 4.3. Leptospira spp. and Seasonality

To date, the role of seasonality in the prevalence of *Leptospira* among wild and synanthropic animals is still not well defined. In our study, seasonality was not significantly related with the prevalence of *Leptospira* among the sampled animals, except in hedgehogs (*p* < 0.05), with a lower prevalence observed during winter, which could be associated with the winter hibernation period. By contrast, a recent study conducted in Spain [21] reported that the *Leptospira* prevalence in peri-urban micromammals ranged from 8% to 13%, and the probability of infection with *Leptospira* was three times higher in spring than in autumn. Other studies have reported a high probability of *Leptospira* detection among susceptible hosts in autumn and have demonstrated that seasonal patterns depend on the region and yearly rainfall in the period under investigation [22,23]. In our study, the non-significance of seasonality in most species could be influenced by the sampling area: in fact, most of the samples belonged to geographical areas in the lower Po Valley, characterized by high humidity levels and by rare extreme winter temperatures [17].

This study assessed the seasonality of Leptospira in red foxes caught predominantly in the alpine region, but no significant differences were found, even if previous studies had described that antibodies against specific serovars were more frequent during the winter period than during the summer [40].

Generally, the present study assessed only the molecular detection of *Leptospira*; furthermore, this survey was conducted on a limited number of animals that certainly does not reflect the size of the animal population present in the studied area. In addition, the data reported in the present study refer to samples of animal carcasses, evaluated within the context of passive surveillance control programs; therefore, it was not possible to standardize sample collection, neither was it possible to collect specific epidemiological and time-related information.

In light of this, it would be useful to envisage monitoring programs for *Leptospira* in wild and synanthropic animals using standardized and repeatable protocols in order to assess prevalence trends among these species.

The present study reported the presence of *Leptospira* strains in wild and synanthropic species, highlighting a possible connection between domestic-synanthropic-wild species and the shared environment. Therefore, the knowledge of regional epidemiology, which can be assessed only by the identification of locally prevalent strains either among domestic animals or wildlife, is necessary to understand infection patterns and transmission chains. Moreover, the lack of knowledge about the correlation between geographical, spatial, and meteorological information needs to be implemented in order to describe possible risk factors in the current climate conditions.

## 5. Conclusions

The study confirmed a broad exposure and geographical distribution of *Leptospira* across synanthropic species and wildlife in northeastern Italy and underlined the relevance of shared environmental exposure among the species. Particularly, *L*. *Australis* ST 24 and *L*. *borgpetersenii* Javanica ST 146 were reported in hedgehogs, mice, and foxes, suggesting the need to further investigate the possible prey–predator epidemiological scenario. Furthermore, *L*. *Australis* ST 198 was detected exclusively in hedgehogs. *L*. *interrogans* Icterohaemorragiae ST 17 was reported to be the most represented in rats, as expected, followed by hedgehogs, mice, and foxes. *L*. *borgpetersenii* Sejroe ST 155 was rarely reported (one hedgehog and one mouse), as well as *L*. *borgpetersenii* Sejroe ST 197 (one vole). *L*. *kirschneri* Pomona Mozdok ST 117 was described in one wolf, suggesting the importance of evaluating the wildlife/domestic interface. In the ecology of leptospirosis and of some other infections given by multi-host pathogens, the definition of reservoir is not totally clear. The detection of a particular ST in animal species belonging to specific ecological contexts (i.e., vole), highlights the need to consider the challenge of characterizing the mechanisms and epidemiological pathways of *Leptospira* exposure or infection in a large number of host animals and the need to define their epidemiological role as environmental sentinel hosts or reservoirs.

## Figures and Tables

**Table 1:** Overall results of the serological survey conducted in coypus in the provinces of Trento and Padua, represented as the number of positive coypus (N), the total number of enrolled coypus (Total), the MAT titre ranges of positive samples, the observed prevalence and the confidence interval of 95% (CI).

Province	Species	Serogroups	Serovar	N	Total	MAT Range	Prevalence (%)	CI
TN	*L interrogans*	Australis	Bratislava	2	30	1:200–1:1600	6.7	0.8–22.1
PD	*L interrogans*	Australis	Bratislava	21	41	1:100–1:3200	51.2	35.1–67.1
	*L interrogans*	Icterohaemorrhagiae	Icterohaemorrhagiae	2		1:100–1:200	4.9	0.0–0.2
	*L interrogans*	Icterohaemorrhagiae	Copenhageni	1		1:100	2.4	0.1–12.9

**Table 2:** Overall results of the animal population enrolled in the study, represented as number of positive and negative subjects, total number of animals enrolled for each species (N), the observed prevalence and the confidence interval of 95% (CI).

Species	Positives	Negatives	N	Prevalence (%)	CI
*Coypus*	9	13	22	40.9	23.2–61.2
*Bank Vole*	3	7	10	30.0	10.8–60.3
*Norway Rat*	25	83	108	23.1	15.6–32.2
*Red Fox*	22	83	105	21.0	13.6–30.0
*Common Shrew*	1	4	5	20.0	1.0–62.4
*Hedgehog*	58	272	330	17.6	13.6–22.1
*House Mouse*	9	70	79	11.4	5.3–20.5
*Greater-Mouse Eared Bat*	1	8	9	11.1	0.6–43.5
*Grey Wolf*	1	12	13	7.7	0.4–33.3
Totals	129	552	681	18.9	16.1–22.1

**Table 3:** Synanthropic and wild animals positive for *Leptospira* spp. and the MLST analysis, reported for each *Leptospira* serogroups. Prevalence and confidence interval (CI) are reported for the Sequence Type found in each species.

Host	ST	Species	Serogroups	Serovar	N	Prevalence	CI 95%
Hedgehog	24	*L interrogans*	Australis	Bratislava/Jalna/Lora/Muenchen	24	54.5%	37.9–68.3
	198			Australis	15	34.1%	20.0–48.9
	17	*L interrogans*	Icterohaemorrhagiae	Icterohaemorrhagiae/Copenhageni	3	6.8%	1.4–11.8
	146	*L borgpetersenii*	Javanica	Javanica/Poi/Sorexjalna	1	2.3%	0.07–11.8
	155	*L borgpetersenii*	Sejroe	Polonica/Saxkoebing/Istrica	1	2.3%	0.07–11.8
House Mouse	24	*L interrogans*	Australis	Australis	1	16.7%	0.9–56.4
	17	*L interrogans*	Icterohaemorragiae	Icterohaemorrhagiae/Copenhageni	3	50%	18.8–81.2
	155	*L borgpetersenii*	Sejroe	Polonica/Saxkoebing/Istrica	1	16.7%	0.9–56.4
	146	*L borgpetersenii*	Javanica	Javanica/ Poi/ Sorexjalna	1	16.7%	0.9–56.4
Norway Rat	17	*L interrogans*	Icterohaemorrhagiae	Icterohaemorrhagiae/Copenhageni	22	100%	0.85–1
Red Fox	24	*L interrogans*	Australis	Bratislava/Jalna/Lora/Muenchen	4	44.4%	18.9–73.3
	17	*L interrogans*	Icterohaemorragiae	Icterohaemorrhagiae/Copenhageni	3	33.3%	12.1–64.6
	146	*L borgpetersenii*	Javanica	Javanica/ Poi/ Sorexjalna	2	22.2%	6.3–54.7
Bank vole	197	*L borgpetersenii*	Sejroe		1	100%	2.6–97.4
Grey Wolf	117	*L. kirschneri*	Pomona	Mozdok	1	100%	5.1–1

ST: Sequence Type. N: number of subjects identified as positive.

**Table 4:** Prevalence of *Leptospira* spp. infection and confidence interval (95%CI) for each season are reported for the overall population and for the hedgehog, Norway rat, house mouse and red fox species. *p* values from the GLMM analysis are reported.

	Spring	Summer	Autumn	Winter	*p*
Hedgehog	14% 95%CI 5.9–27.2	25.3% 95%CI 16.6–35.7	17.6% 95%CI 11.7–24.9	7.7% 95%CI 2.1–18.5	*p* = 0.04
Norway Rat	28.6% 95%CI 15.7–44.6	16.7% 95%CI 0.4–64.1	17.8% 95%CI 8.0–32.1	26.7% 95%CI 7.8–55.1	*p* = 0.7
House Mouse	5.9% 95%CI 0.7–19.7	22.7% 95%CI 7.8–45.4	9.1% 95%CI 0.2–41.3	8.3% 95%CI 0.2–38.4	*p* = 0.8
Red Fox	25% 95%CI 8.7–49.1	18.2% 95%CI 5.2–40.3	11.1% 95%CI 1.4–34.7	24.4% 95%CI 12.9–39.5	*p* = 0.7
Overall	20% 95%CI 14.3–26.6	24% 95%CI 17.3–31.4	17% 95%CI 12.3–22.6	15.2% 95%CI 9.6–22.6	*p* = 0.4

## Data Availability

All the data available are reported within the manuscript.
